# Synergistic potential of Leu_10_-teixobactin and cefepime against multidrug-resistant *Staphylococcus aureus*

**DOI:** 10.1186/s12866-024-03577-x

**Published:** 2024-10-29

**Authors:** Augustine Jing Jie Koh, Maytham Hussein, Varsha Thombare, Simon Crawford, Jian Li, Tony Velkov

**Affiliations:** 1 Department of Biochemistry and Pharmacology, School of Biomedical Sciences, Faculty of Medicine, Dentistry and Health Sciences, The University of Melbourne, Parkville, VIC 3010 Australia; 2 Monash Biomedicine Discovery Institute, Department of Pharmacology, Monash University, Clayton, VIC 3800 Australia; 3 Monash Biomedicine Discovery Institute, Department of Biochemistry and Molecular Biology, Monash University, Clayton, VIC 3800 Australia; 4 Monash Biomedicine Discovery Institute, Department of Microbiology, Monash University, Clayton, VIC 3800 Australia

**Keywords:** Leu_10_-teixobactin, Cefepime, Methicillin-resistant *Staphylococcus aureus* (MRSA), Synergistic activity, Biofilm inhibition, β-lactam potentiation

## Abstract

*Staphylococcus aureus* (*S. aureus*) is a significant Gram-positive opportunistic pathogen behind many debilitating infections. β-lactam antibiotics are conventionally prescribed for treating *S. aureus* infections. However, the adaptability of *S. aureus* in evolving resistance to multiple β-lactams contributed to the persistence and spread of infections, exemplified in the emergence of methicillin-resistant *S. aureus* (MRSA). In the present study, we investigated the efficacies of the synthetic teixobactin analogue, Leu_10_-teixobactin, combined with the penicillinase-resistant cephalosporin cefepime against MRSA strains. The Leu_10_-teixobactin and cefepime combination exerted synergism against most strains tested in broth microdilution assay. Time-kill profiles showed that both Leu_10_-teixobactin and cefepime predominantly exhibited synergistic activity, with > 2.0-log_10_CFU decrease compared to monotherapy at 24 h. Moreover, biofilm assays revealed a significant inhibition of biofilm production in ATCC™43300 cells treated with sub-MICs of Leu_10_-teixobactin and cefepime. Subsequent electron microscopy studies showed more extensive damage with the combination therapy compared to monotherapies, including aberrant bacterial morphology, vesicle formation and substantial lysis, indicating combined damage to the cell wall. Quantitative real-time PCR revealed marked perturbation of genes *mecA*, *sarA*, *atlA*, and *icaA*, substantiating the apparent mode of combined antibacterial action of both antibiotics against peptidoglycan synthesis and initial biofilm production. Hence, the study highlights the prospective utility of the Leu_10_-teixobactin-cefepime combination in treating MRSA infections via β-lactam potentiation.

**Supplementary Information:**

The online version contains supplementary material available at 10.1186/s12866-024-03577-x.

## Introduction

The Gram-positive bacterium *S. aureus* causes a broad range of infections, from mild skin infections like boils and abscesses to severe complications including wound infections, UTIs, pneumonia, meningitis, endocarditis, toxic shock syndrome, bacteremia, and sepsis [1]. Despite the development of penicillinase-resistant β-lactam penicillin derivatives (e.g., methicillin, oxacillin) to combat penicillin-resistant *S. aureus*, resistance still rapidly developed and led to the rise of methicillin-resistant *S. aureus* (MRSA) [2, 3]. Moreover, MRSA is highly capable of biofilm production conferring augmented resistance to antimicrobials, virulence, and protection from the immune system [4–6].

The primary inhibitory target for β-lactam antibiotics in *S. aureus* is penicillin-binding protein 2 (PBP2). MRSA expresses PBP2a that is expressed by the mecA gene located on the staphylococcal cassette chromosome mec (SCCmec) mobile genetic element [7, 8]. Compared to PBP2, PBP2a has a lower binding affinity to many β-lactam antibiotics, including fourth-generation cephalosporin cefepime, preventing the inhibition of peptidoglycan chain crosslinking (i.e., transpeptidation) [9–13]. Worryingly, the recent report by the WHO in 2024 listed *S. aureus* as a priority pathogen requiring urgent treatment strategies [14]. This warrants the development of stronger β-lactam classes or alternate cell wall antibiotics targeting different precursors of peptidoglycan synthesis [7, 15].

Teixobactin was first extracted from the soil bacterium *Eleftheria terrae* and was observed to exhibit efficacious anti-Gram-positive activity [16, 17]. A non-ribosomal macrocyclic peptide that binds lipid II, inhibiting peptidoglycan polymerization (i.e., transglycosylation) [18]. Multiple analogues of teixobactin have been synthesized, one of which is Leu_10_-teixobactin (with Leucine substituting L-allo-enduracididine as the 10th amino acid residue), which has emerged as an experimental alternative to teixobactin due to its ease of synthesis, and exhibition of bactericidal activity comparable to that of native teixobactin [18–20].

The present study aims to investigate the potential synergy for Leu_10_-teixobactin (L_10_TXB) in combination with the 4th -generation cephalosporin cefepime against a range of *S. aureus* strains, including MRSA. Additionally, it explores the mechanisms of synergistic action using electron microscopy and quantitative real-time PCR to assess expressions of select genes involved in peptidoglycan production and biofilm formation.

## Results

### Broth microdilution: antibacterial synergy of Leu10-teixobactin and cefepime in combination

The antimicrobial activity of Leu_10_-teixobactin (L_10_TXB) and cefepime (CEF), alone and combined, were tested against a panel of seven *S. aureus* strains with varying degrees of β-lactam resistance [21]. According to the European Committee on Antimicrobial Susceptibility Testing (EUCAST), cephalosporin susceptibility (and resistance) was inferred from the cefoxitin breakpoints, with MIC resistance defined as > 4 µg/mL [22]. Most of the strains tested exhibited cefepime MICs > 4 µg/mL, verifying general resistance to cephalosporins. The L_10_TXB/CEF combination exerted synergy against most of the strains assayed (Table 1), with particularly heightened synergy (FIC_Index_ 0.266–0.375) observed with ATCC™29213, ATCC™43300, ATCC™700698, and JKD6159. Only additivity (FIC_Index_ 0.75) and indifferentism (FIC_Index_ 2.00) were observed in JKD6008 and JKD6009 respectively. Static time-kill assay was conducted next to confirm synergistic activity [23].

**Table 1:** Antimicrobial activity of Leu_10_-teixobactin (L_10_TXB), and cefepime (CEF) monotherapy, and in combination. MIC – minimal inhibitory concentration. FIC_Index_ – fractional inhibitory concentration index: ***FIC***_***Index***_***≤0.5 (synergy)***, > 0.5-1.0 (additivity), > 1–4 (indifference), ˃4 (antagonism). S – susceptible, R – resistant

Bacteria	Strain	MRSA	Cefepime susceptibility	Monotherapy MIC (µg/mL)		Combined MIC (µg/mL)		FIC_Index_
				L_10_TXB	CEF	L_10_TXB (+ CEF)	CEF (+ L_10_TXB)	
*S. aureus*	ATCC™29213	No	S	1	4	0.125	1	***0.375***
	ATCC™43300	Yes	R	1	128	0.25	2	***0.266***
	ATCC™700698			1	> 256	0.25	64	***0.375***
	ATCC™700699			2	256	0.5	64	***0.500***
	JKD6009			0.25	> 256	0.25	> 256	2.000
	JKD6008			1	256	0.25	128	0.750
	JKD6159			1	64	0.25	4	***0.375***

### Static time-kill studies

**Fig. 1:**
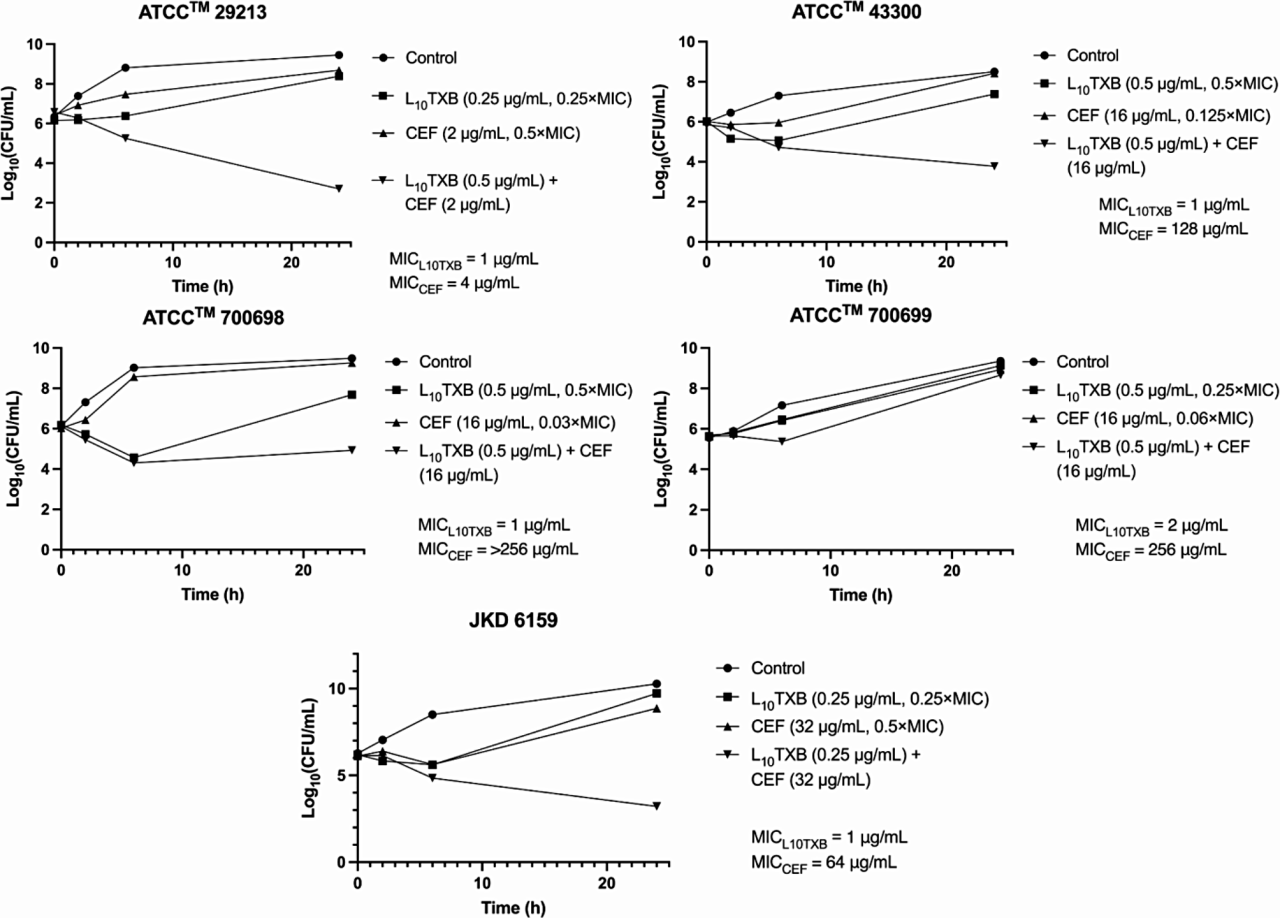
Standard inoculum time-kill graphs (Initial inoculum/strain log_10_CFU/mL ~ 6.0) of Leu_10_-teixobactin (L_10_TXB) and cefepime (CEF) alone and in combination against *S. aureus* ATCC™29213, ATCC™43300, ATCC™700698, ATCC™700699, and JKD6159. Bacterial counts are presented as the mean from two independent repeats

The killing kinetics of the L_10_TXB/CEF combination were assessed against the methicillin-susceptible strain ATCC™29213 (MIC_L10TXB_ = 1 µg/mL; MIC_CEF_ = 4 µg/mL) and the methicillin-resistant strains ATCC™43300 (MIC_L10TXB_ = 1 µg/mL; MIC_CEF_ = 128 µg/mL), ATCC™700698 (MIC_L10TXB_ = 1 µg/mL; MIC_CEF_ = > 256 µg/mL), ATCC™700699 (MIC_L10TXB_ = 2 µg/mL; MIC_CEF_ = 256 µg/mL), and JKD6159 (MIC_L10TXB_ = 1 µg/mL; MIC_CEF_ = 64 µg/mL) with final antibiotic concentrations equivalent to the FICs in checkerboard (Fig. 1) [24]. Due to the time-dependent activity of L_10_TXB, the peptide was observed to primarily drive combination activity at 6 h post-treatment. Additive antibacterial activity was observed in combination-treated ATCC™700699 (> 1.1-log_10_CFU decrease or difference in the colony counts relative to L_10_TXB monotherapy at 6 h) and ATCC™700698 bacteria (-1.2Δlog_10_CFU compared to the initial inoculum at 24 h), likely in part, due to having thicker cell walls [8, 25]. At 24 h, the combination-treated bacteria of three of the strains (ATCC™29213, ATCC™43300, and JKD6159) exhibited a > 2.0-log_10_CFU decrease in viability from the initial inoculum compared to L_10_TXB monotherapy, with bactericidal activity (>-3.8Δlog_10_CFU compared to the initial inoculum) observed in combination-treated ATCC™29213 bacteria (Table S1).

### Electron microscopy (EM) imaging of MRSA treated with Leu10-teixobactin and cefepime in combination

**Fig. 2:**
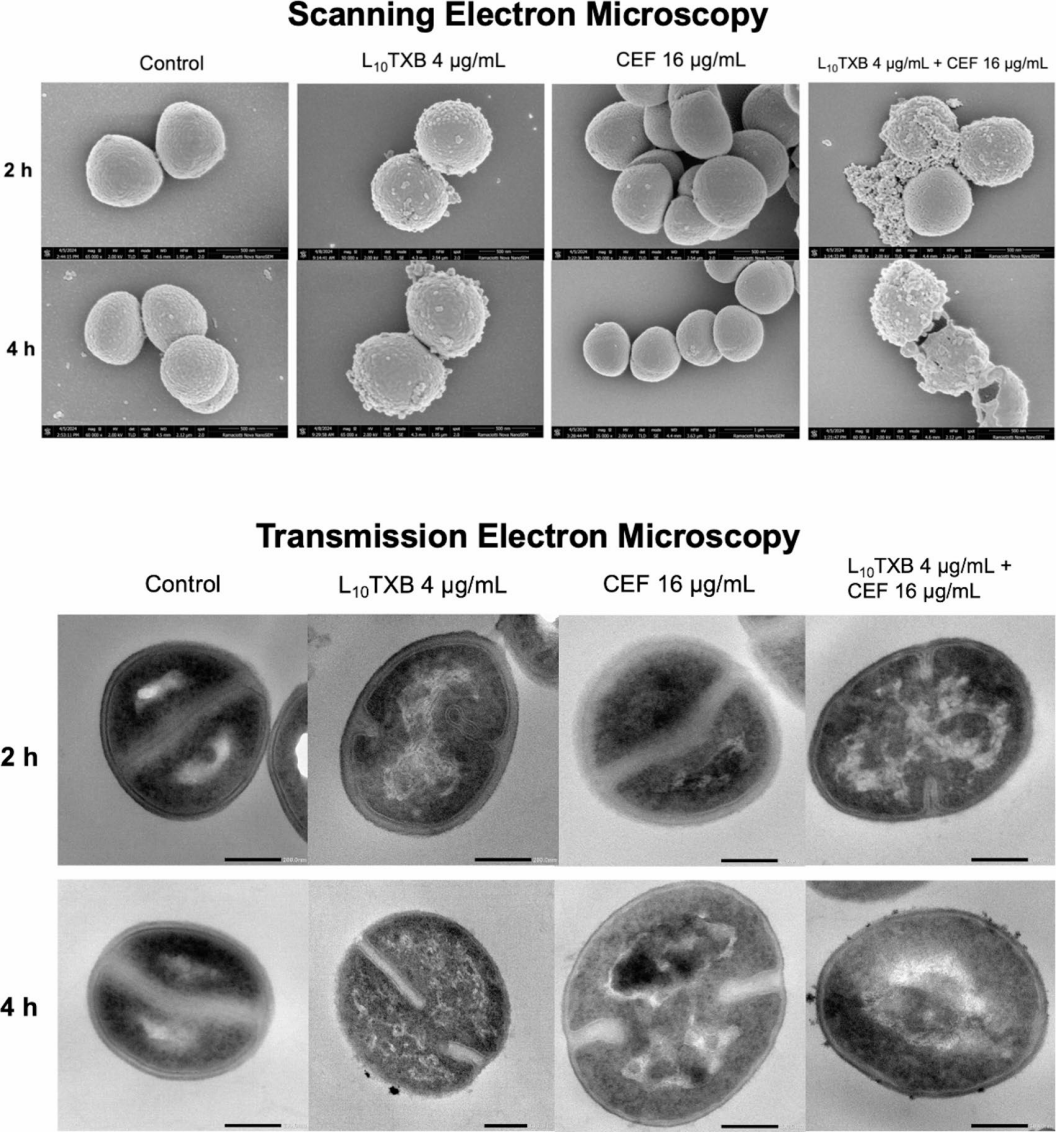
Scanning electron microscopy and Transmission electron microscopy images of ATCC™43300, treated with Leu_10_-teixobactin (L_10_TXB, 4 µg/mL) and cefepime (CEF, 16 µg/mL), alone and in combination at 2 h and 4 h post-treatment

Scanning electron microscopy (SEM) and transmission electron microscopy (TEM) imaging techniques were carried out to understand the impact of the Leu_10_-teixobactin/cefepime combination on the bacterial cell wall [26]. The MRSA strain ATCC™43300 (MIC_L10TXB_ = 1 µg/mL; MIC_CEF_ = 128 µg/mL) was treated with both antibiotics, alone and in combination, with samples aliquoted at 2 h and 4 h post-treatment for fixing and subsequent imaging (Fig. 2). The cefepime-treated bacteria predictably displayed intact morphology and cell envelopes akin to the untreated bacteria due to high β-lactam resistance. Despite the intact morphology observed in L_10_TXB-treated bacteria, initial blebbing was seen at 2 h which became more pronounced at 4 h with protrusions and apparent vesicle formation suggesting membrane bilayer damage, concordant with the time-dependent activity of the peptide. The combination-treated bacteria at both time points displayed irregular morphology, shrinkage, and perforation of the cell envelope compared to either monotherapy where the damage to the cell envelope was minimal, suggesting augmented damage to the peptidoglycan cell wall and impacted cell division.

The SEM images were further corroborated by the TEM images. At 2 h, the monotherapy-treated bacteria displayed and septal formation aligned at the cell equator, indicative of regular binary fission. However, following combination-treatment, the bacterium exhibited membrane invaginations forming away from the cell equator, leaving one budding daughter bacterium of irregular size than the other. Concordant with time-dependent activity, the L_10_TXB-treated bacterium revealed conspicuous blebbing and vesicle formation at 4 h compared to 2 h. Notably, greater vesicle formation was observed in the combination-treated bacterium at 4 h post-treatment, indicating augmented damage to the cell envelope. As expected, between 2 h and 4 h, the cefepime-treated bacterium exhibited insignificant changes in morphology and cell envelope thickness relative to the untreated bacterium.

### Combined effect of Leu10-teixobactin and cefepime on biofilm production

**Fig. 3:**
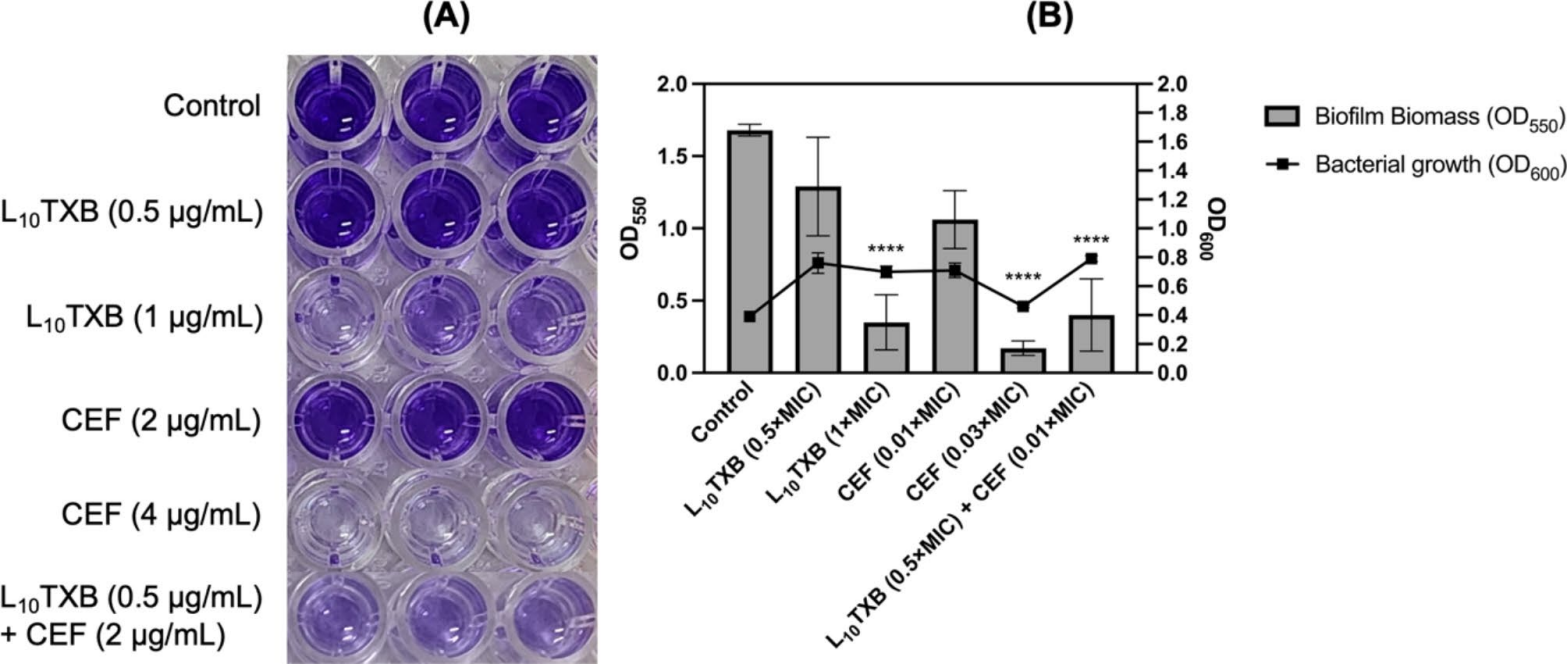
Effect of Leu_10_-teixobactin (L_10_TXB) and cefepime (CEF) alone and in combination on the formation of MRSA ATCC™43300 biofilms via crystal violet quantification (**A**). The columns and the lines (**B**) represent the average biofilm biomass and bacterial growth of triplicate experiments respectively. The error bars exemplify standard deviation. The asterisks show the statistical difference between the treatment groups and the untreated control, determined by one-way ANOVA followed by Tukey’s honest significant test. Significance was accepted when the *p*-value was < 0.05 (****p < 0.001)

The ATCC™43300 cells were incubated for 24 h in 3% glucose-supplemented TSB in the absence and presence of subinhibitory concentrations of Leu_10_-teixobactin and cefepime, *per se*, and combined. Compared to the untreated control, the crystal violet strain showed both L_10_TXB (1 µg/mL, 1×MIC) and cefepime (4 µg/mL, 0.03×MIC) monotherapies capable of substantially inhibiting biofilm formation (~ 80–90% reduction of biofilm biomass, *p* < 0.001) compared to L_10_TXB (0.5 µg/mL, 0.5×MIC) and cefepime (2 µg/mL, 0.01×MIC) (~ 23% and ~ 37% reduction respectively) (Fig. 3A). Notably, the combination of L_10_TXB (0.5×MIC) and cefepime (0.01×MIC) inhibited biofilm production extensively by ~ 76% (*p* < 0.001) than the use of the same concentrations of either drug alone (~ 30–40% biofilm biomass reduction) (Fig. 3B).

### Significant perturbation of cell wall and biofilm gene expression by Leu10-teixobactin and cefepime combined

**Fig. 4:**
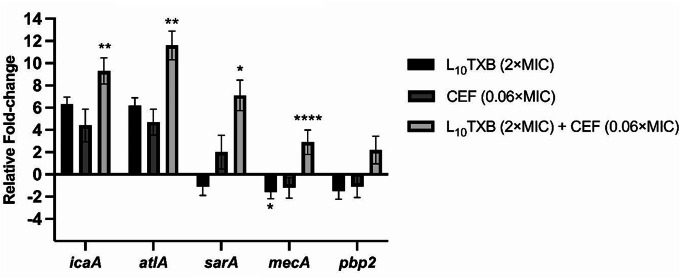
Real-time quantitative PCR analysis of *icaA*, *atlA*, *sarA*, *mecA*, and *pbp2* gene transcription in log-phase ATCC™43300 at 4 h post-treatment with Leu_10_-teixobactin and cefepime, alone and in combination. Gene expression levels were normalized to the 16S rRNA gene. The asterisks represent the statistical difference between the treatment groups and the untreated control, determined by one-way analysis of variance (ANOVA) followed by Tukey’s honest significant test. Significance was accepted when the *p*-value < 0.05* (****p-value < 0.0001)

A total of five genes functionally contributing to β-lactam resistance, late-stage peptidoglycan synthesis, and biofilm formation (Table S2) were selected to determine the transcriptional response of MRSA ATCC™43300 in exponential growth to Leu_10_-teixobactin and cefepime treatment, alone and in combination. The expression of *pbp2* was observed to be upregulated (~ 3.5-fold) in combination-treated bacteria than monotherapy-treated bacteria (~ 1.7-1.8-fold) but not statistically significant compared to untreated bacteria. The level of *mecA* expression was extensively decreased during 2×MIC L_10_TXB (*p* < 0.05) treatment whereas treatment with both 2×MIC L_10_TXB and 0.06×MIC CEF substantially increased expression (> 2.0-log_2_FC) with respect to the untreated control. The expression of the *atlA* gene (> 10.0-log_2_FC) was substantially increased compared to either 2×MIC L_10_TXB or 0.06×MIC CEF monotherapy relative to control (*p* < 0.0001). Similarly, the upregulation of *sarA* and *icaA* was significantly higher with the combinatorial treatments than with L_10_TXB or CEF at the same concentrations alone (*p* < 0.0001) (Fig. 4).

## Discussion

Generally, treatment of MRSA-related infections is challenging due to the production of structurally modified PBPs and biofilm generation. Cephalosporins are most commonly used β-lactam antibiotics clinically, but the adaptability of *S. aureus* has enabled the rapid acquisition of resistance [27]. Thus, one of the most effective therapeutic approaches is to use combinatorial antimicrobial therapies to potentiate β-lactam activity against such infections [28, 29]. Here, we examined the antibacterial effects of combining the antimicrobial peptide Leu_10_-teixobactin and the cephalosporin cefepime on MRSA. Teixobactin can inhibit synthesis of wall teichoic acids (WTA) in addition to its effect on transglycosylation [16, 30]. Prior studies have shown that WTAs are needed for the proper localization, orientation and coordination of PBP2a, PBP2 and PBP4 for optimal transpeptidation. MRSA strains with blocked WTA synthesis (i.e., knockout mutants and regular strains treated with WTA blockers) were found to display weakened peptidoglycan crosslinking, septal defects and re-sensitization to β-lactams [31–34]. For this reason, we postulated that teixobactin can potentiate cefepime against MRSA; exerting augmented combined damage to the peptidoglycan cell wall. This was supported by the predominantly synergistic activity observed in broth microdilution and time-kill assays. The SEM and TEM images further supported the synergy, revealing extensive shrivelling, vesicle formation, and lysis in combination-treated MRSA compared to either agent alone, suggesting the weakening of the cell envelope as the synergistic mechanism of combined antibiotic action.

A recent study revealed the capability of various teixobactin analogues in eradicating *S. aureus* biofilms [35]. WTAs constitute the most abundant component of the *S. aureus* cell wall and are involved in surface adhesion and colonization during biofilm formation. This likely explains the observed inhibition of biofilm production following Leu_10_-teixobactin treatment (1 µg/mL, 1×MIC) in the biofilm assay [36–38]. Additionally, a previous study confirmed the ability of cephalosporins to inhibit *S. aureus* biofilm production but did not investigate the possible mechanisms behind biofilm inhibition [27]. Our anti-biofilm assay findings similarly supported these results, showing that both Leu_10_-teixobactin and cefepime could substantially inhibit biofilm formation when combined at subinhibitory concentrations.

The real-time PCR showed significant upregulation of *sarA* and *icaA* gene expression in combination-treated cells. The staphylococcal accessory regulator (SarA) protein is an important positive regulator of biofilm formation and functions as a transcription factor expressing the *icaA* gene, leading to the production of poly-N-acetylglucosamine which is vitally involved in initial bacterial adhesion and aggregation – the first phase of biofilm production [39, 40]. This suggests substantial perturbation of poly-N-acetylglucosamine synthesis by both antibiotics, implicating initial bacterial adhesion. Hence, the observed heightened expression of both genes was likely to mitigate the combined antibiofilm effects of both antibiotics [41–43].

A previous study involving another Gram-positive bacterium, *Enterococcus faecalis*, reported the perturbation of several penicillin-binding protein genes post-treatment with high teixobactin concentrations [44]. Cefepime is already known to exhibit high affinity for PBP2 and also targets PBP3 and PBP1 in *S. aureus* [32, 45]. This supports our findings that both cefepime and Leu_10_-teixobactin monotherapies dysregulated the expression of the *pbp2* gene. Challenging Gram-positive bacteria with cell wall-targeting antibiotics causes nutrient deprivation, oxidative and cell envelope stress; thus, eliciting regulated changes in the expression of specific genes as a defensive means of survival and maintaining homeostasis [44, 46, 47]. Hence, this could rationalise the over-expression of the *pbp2* and *mecA* following the Leu_10_-teixobactin-cefepime combination therapy to sustain transpeptidation.

Autolysins are endogenous hydrolases involved in peptidoglycan recycling to facilitate *S. aureus* growth and division for survival [48, 49]. The heightened expression of *atlA* in combination-treated bacteria was likely to expedite autolysin production for peptidoglycan remodeling and binary fission, which unintentionally exacerbates peptidoglycan degradation and leading to apparent irregularly-sized daughter bacteria and lysed bacteria in electron microscopy. Overall, our data demonstrates the potential for Leu_10_-teixobactin and cefepime antibiotic combination as a potent antimicrobial and antibiofilm agents against MRSA, suggesting its future applications as a novel approach to eliminate problematic biofilms and associated infections, including MRSA infections.

## Materials and methods

### Synthesis of Leu_10_-teixobactin

Synthesis was undertaken using 2-Chlorotrityl Chloride Resin (0.1 mmol) (100–200 mesh 0.4-1.0 mmol/g). The first amino acid (Leu_10_) was loaded using a 6 molar equivalents solution of Fmoc-amino acid in DMF. The rest of the amino acids from **1** to **10** were coupled via the Protein Technologies Prelude automated peptide synthesizer using standard Fmoc solid-phase peptide chemistry. Coupling of the Fmoc-amino acids was performed using the default instrument protocol: 3 molar equivalents (relative to resin loading) of the Fmoc amino acid and HCTU in DMF with activation in situ, using 6 molar equivalents of DIPEA. This was carried out for 50 min at room temperature. Fmoc deprotection was conducted using the default instrument protocol: 20% piperidine in DMF (1 × 5 min, 1 × 10 min) at room temperature. The synthesized peptide (**1-10**) resin was dried overnight. In another flask, the Fmoc-Ile-OH (5 equivalent) was dissolved in dry DMF under nitrogen, then 5 equivalent DIC was added dropwise at 0^o^C, and the mixture was stirred for 5 min. After the activation of Fmoc-Ile-OH acid, the overnight dried resin was transferred into the solution, 0.3 equivalent DMAP was added, and the reaction mixture was stirred overnight.

The synthesized linear peptide resin was utilized before the deprotection of the Ile_11_ Fmoc protecting group by 20% piperidine for 5 min twice. The protected linear peptide was then cleaved from the resin by treating the resin with 20% hexafluoroisopropanol (HFIP) in DCM (1 × 30 min, 1 × 5 min). This solution was concentrated under a vacuum to give the crude-protected linear peptide. The protected linear peptide was dissolved in DMF (5 mL) to which DIPEA 0.6 mmol, 104 µL (6 molar equivalents relative to the loading of the resin) and DPPA, 0.3 mmol, 0.65 µL (3 molar equivalents relative to the loading of the resin) were added. The solution was shaken overnight at room temperature. The reaction solution was then concentrated under a vacuum for a minimum of 6 h to give the crude-protected cyclic peptide. The resulting residue was taken up in a solution of 2.5% DODT and 5% TIPS in TFA and stirred at room temperature for 90 min. To this solution, 40 mL of diethyl ether was added. The precipitate was centrifuged and washed twice with diethyl ether (40 mL), then air-dried in a fume hood to give the crude cyclic peptide (a pale-yellow solid). The resulting solid was taken up in Milli-Q water (5 mL) and de-salted using a Vari-Pure IPE SAX column. The crude cyclic lipopeptide was then subjected to RP-HPLC. L_10_TXB (Fig. S1A) was obtained in a yield of 7.4 mg, retention time (R_t_) at 214 nm = 15.23 min (purity: 94.5%). ESI-MS analysis of peak at 15.23 min: m/z (monoisotopic) [M + 1 H]^1+^ 1202.10 [M + 2 H]^2+^ 601.85. Calculated mass (monoisotopic) for Teixobactin (C_58_H_96_N_12_O_15_) 1201.71 (Fig. S1B).

### Bacterial strains

Seven *S. aureus* strains, four of which were supplied from the American Type Culture Collection (ATCC™), were selected for the study and stored in tryptone soy broth (Oxoid) with 20% glycerol (Ajax Finechem, Seven Hills, NSW, Australia) in cryovials (1.8 mL each) kept at -80 °C [50]. Six MRSA strains were sub-cultured and incubated at 37 °C overnight, ATCC™43300 and JKD6009 (vancomycin-susceptible), ATCC™700698 (heterogenous vancomycin-intermediate), ATCC™700699 and JKD6008 (vancomycin-intermediate), and JKD6159 (community-associated MRSA). The methicillin-susceptible *S. aureus* strain ATCC™29213 (mecA-negative, penicillinase-positive) serves as the quality control strain [8, 25, 51–53].

### Broth checkerboard microdilution

Stock antibiotic solutions (5120 µg/mL) of Leu_10_-teixobactin and the 4th generation cephalosporin cefepime (CGene Tech Inc) were prepared immediately before each experiment. MIC (minimal inhibitory concentration) and FIC (fractional inhibitory concentration) assaying were conducted in triplicate according to the CLSI-M100 guidelines [51]. The CAMHB (Oxoid, England) media was supplemented with 0.1% v/v polysorbate-80 (Selleckchem) to prevent adhesion of Leu_10_-teixobactin to the surface of the wells in the 96-well microtiter plates (Techno Plas) [50]. If no endpoint β-lactam MIC value could be determined because of high resistance, the next highest MIC was chosen for the FIC calculation (e.g., MIC = 512 µg/mL if MIC > 256 µg/mL). The FIC index (FIC_Index_) is calculated through the following equation (MIC_Combined_ L_10_TXB ÷ MIC_L10TXB_) + (MIC_Combined_ Cefepime ÷ MIC_Cefepime_), and antimicrobial interactions were classified as synergism (FIC_Index_ ≤0.5), additivity (FIC_Index_ >0.5-1.0), indifference (FIC_Index_ >1–4) and antagonism (FIC_Index_ ˃4) [54–56].

### Static time-kill assay

A single colony was aseptically inoculated into CAMHB (10 mL) in a 50-mL Falcon tube (ThermoFisher Scientific, Australia) for incubation overnight (~ 16–18 h) at 37 °C in the orbital shaker-incubator at 180 rpm (New Brunswick Scientific) [20, 57]. The overnight culture (ONC) was diluted 100-fold in pre-warmed CAMHB (10 mL) and incubated in the shaker (37 °C) for at least 2 h to reach ~ 10^8^ CFU/mL. Next, the log culture is diluted 100-fold in pre-warmed CAMHB (20 mL in each 50-mL borosilicate glass tube) to yield an initial inoculum ~ 10^6^ CFU/mL. Three of the tubes were treated with antibiotics *per se* and in combination, yielding final concentrations of cefepime, equivalent to therapeutic plasma concentrations below the toxicity threshold (i.e., 35 µg/mL), and 0.25–0.5×MIC for L_10_TXB [58–60]. The remaining treatment tube serves as the antibiotic-free control.

Aliquots (200 µL) were removed from the treatment tubes at designated time points and serially diluted in 0.9% w/v saline in 5-mL dilution vials (Techno-Plas, 1.8 mL/vial). The bacterial suspensions from the dilution vials were streaked onto Nutrient agar (Oxoid) plates (50 µL/plate), manually under aseptic conditions or via the Don Whitley automated spiral plater, for overnight incubation to determine bacterial viability at differing incubation time points via colony count (i.e., colony-forming units, CFU/mL). Time–kill curves were constructed by plotting mean colony count (log_10_CFU/mL) against time. Bactericidal activity is defined as a > 3-log_10_CFU reduction in bacterial viability at any time from the initial inoculum while bacteriostatic activity is defined as a > 0 to < 3-log_10_CFU reduction from the initial inoculum [61, 62]. Synergism is defined as a ≥ 2-log_10_CFU-reduction in the bacterial counts between the combination and the more active antibiotic in the combination, and with the population of viable bacteria to be ≥ 2-log_10_CFU below the starting inoculum at any investigated time-point [63–65]. Additivity is defined as a > 1- to < 2-log_10_CFU reduction in the colony counts for the combination compared with the most active antibiotic, and antagonism as a regrowth to ≥ 1-log_10_CFU for the combination relative to the least active antibiotic [66].

### Scanning and transmission electron microscopy

A single colony of ATCC™43300 was aseptically inoculated into CAMHB (10 mL) in a 50-mL Falcon tube (ThermoFisher Scientific, Australia) for incubation overnight (~ 18 h) at 37 °C in the shaker-incubator at 180 rpm (New Brunswick Scientific). The following day, the ONC was diluted 100-fold in four separate 50-mL Falcon tubes containing CAMHB (20 mL), leaving one as the untreated control. The tubes were incubated in the shaker incubator for approximately 2.5 h to generate turbid log-phase bacterial cultures (~ 10^8^ CFU/mL). Leaving the untreated control tube, the remaining tubes were treated with Leu_10_-teixobactin and cefepime yielding final concentrations of 4 µg/mL and 16 µg/mL respectively, *per se* and in combination. After the respective treatment incubation times (i.e., 2 and 4 h), the bacteria were pelleted by centrifugation (3220×g, 10 min), and the pellets were washed twice with phosphate-buffered saline (PBS, 5 mL/ tube). The pellets were resuspended in 1 mL PBS for transfer into separate 2-mL Eppendorf tubes and centrifuged (3220×g, 10 min) to fully wash off residual antibiotics. 2.5% glutaraldehyde in PBS (0.5 mL/ tube) was added to the pellets in the tubes, which were placed on the rocker shaker for 0.5–1 h (700 rpm) at room temperature for cell fixing. After cell fixing, the tubes were centrifuged (3220×g, 10 min) and the pellets were washed with PBS twice (1 mL/ tube), followed by resuspension of pellets in 0.5–1.5 mL PBS depending on the pellet size yielded.

For scanning electron microscopy, aliquots of the fixed cells (200 µL each) were incubated on 22 mm glass coverslips coated with 0.1% polyethyleneimine for 30 min. The fixed cells on coverslips were gently rinsed in milli-Q water by immersion for 30 s. Cells on coverslips were dehydrated by incubating in increasing concentrations of ethanol for 15 min each step, consisting of 30, 50, 70, 90, and 2 × 100% ethanol. The dehydrated cells were dried in a Leica EM CPD 300 critical point drier. The coverslips were mounted on 25 mm diameter aluminium SEM stubs using sticky carbon tabs. Mounted coverslips were gold-coated with a Bal-Tec SCD 005 sputter coater. The cells adhered to the coverslips were imaged with a Thermo-Fisher Nova NanoSEM 450 scanning electron microscope at a voltage of 2 kV and a spot size of 2 [67].

For Transmission electron microscopy, dehydration of the fixed cell pellets was conducted via incubation in ethanol incrementally (30, 50, 70, 90, 100%) for 15 min. The ethanol was substituted with 100% propylene oxide. The pellets were incubated in a 1:1 Epon resin/propylene oxide mixture for 6 h at room temperature, followed by overnight incubation in a 2:1 Epon/propylene oxide mix. The bacteria were incubated in 100% freshly-made Epon resin for 6 h, and overnight next in another 100% freshly-made Epon resin. The pellets were inserted into Beem capsules in 100% resin placed in an oven for polymerization (48 h, 60 °C). The resin-embedded tissue was subsequently sectioned with a Diatome diamond knife using a Leica UCS ultramicrotome. 70–90 nm sections of thickness were collected onto formvar-coated 100 mesh copper grids and stained sequentially with 1% uranyl acetate for 10 min and lead citrate for 5 min. The sections were imaged in a JEOL 1400 + transmission electron microscope at 80 kV, and images were captured with a digital camera at a resolution of 2 K x 2 K [68].

### Biofilm inhibition Assay (Crystal Violet Stain)

Stock Leu_10_-teixobactin and cefepime solutions were serially diluted 2-fold in Tryptone Soy Broth (TSB, Merck), supplemented with 3% v/v glucose (Sigma-Aldrich) in 5-mL dilution vials (Techno-Plas), generating concentrations from 8 µg/mL to 2 µg/mL. To assess biofilm inhibitory properties of antibiotics *per se* and in combination, the antibiotics are dispensed into the wells (50 µL/ well) of the flat-bottomed 96-well plates (Falcon^®^). Colonies of ATCC™43300 were inoculated in 0.9% w/v saline and measured via the McFarland meter (bioMérieux) to reach the 0.5 McFarland turbidity standard (~ 10^8^ CFU/mL). The resulting bacterial saline suspension (100 µL) was then diluted 100-fold in 3% v/v glucose-supplemented TSB (10 mL) to yield a starting inoculum of approximately 10^6^ CFU/mL for dispensing into flat-bottomed 96-well plates (100 µL/well) for 24 h incubation. Thus, the final antibiotic concentrations in the wells were quartered; the wells containing single antibiotics were topped up with 3% glucose-supplemented TSB (50 µL/well). Bacterial enumeration was determined via optical density (OD) measurement at 600 nm via the SPECTROstar^®^ Nano Microplate reader.

Planktonic cells are washed out with phosphate-buffered saline (PBS, 220 µL/well) thrice. 0.1% w/v crystal violet solution was dispensed into the wells (220 µL/well) and left at room temperature for 30 min. The crystal violet solution was discarded and the plates were rinsed with PBS thrice. Following drying of the microplates, the biofilms are then solubilized with 30% v/v acetic acid (220 µL/well) for 30 min at room temperature. For the measurement of changes in biofilms, the contents were transferred to a new flat-bottomed 96-well plate (200 µL/well). Absorbance values at 550 nm were obtained using the SPECTROstar^®^ Nano Microplate reader. A one-way analysis of variance (ANOVA) test (concurrent with Tukey’s honest significant test) was used to compare differences between the control and antibiotic-treated biofilms. A *p*-value of < 0.05 was deemed statistically significant.

### Quantitative real-time PCR: gene expression and statistical analysis

Briefly, an overnight culture of ATCC™43300 in TSB with 3% v/v glucose (200 µL) was diluted 100-fold in four TSB with 3% v/v glucose (20 mL) to grow to mid-logarithmic phase (OD_600_ 0.2, ~10^6^ CFU/mL). Three biological replicates were prepared. Following treatment with Leu_10_-teixobactin (2 µg/mL) and cefepime (8 µg/mL) alone and in combination, the tubes were incubated for 4 h and subsequently centrifuged (3220×g, 10 min). Total RNA was extracted from the ATCC™43300 cell pellets using an RNA extraction kit (Qiagen RNeasy Mini Kit) and converted to cDNA following the manufacturers’ protocol from the QuantiTect Rev. Transcription Kit. The PCR amplification was conducted following the QuantiNova SYBR Green PCR Kit manufacturers’ instructions (Qiagen). A 20-µL reaction mixture was prepared containing 10 µL of 2× QuantiNova SYBR green PCR master mix, 0.7 µM of forward and reverse primer each (Table S2), and 2 µL of cDNA sample to be amplified using the AriaMx real-time PCR system (Agilent Technologies). The 16S rRNA gene was used as the housekeeping gene, and the comparative threshold cycle (ΔΔC_T_) method was used to analyze the relative expression levels of the targeted gene transcripts [69].

All experiments were performed in three biological replicates, and results were expressed as mean ± SD. Statistical analyses were performed using GraphPad Prism version 10.2.3 for macOS (GraphPad Software, La Jolla, California, USA). ANOVA followed by Tukey’s honest significant test was used to compare differences in gene expression levels between the antibiotic-treated samples and the untreated control. All statistical analyses were done with a confidence level of 95%, and a *p*-value of < 0.05 was considered statistically significant.

## Electronic supplementary material

Below is the link to the electronic supplementary material.

Supplementary Material 1

## Data Availability

No datasets were generated or analysed during the current study.
