# Supplementary material for: Synergistic potential of Leu10-teixobactin and cefepime against multidrug-resistant Staphylococcus aureus
Source: BMC Microbiol. 2024 Oct 29;24:442. doi: 10.1186/s12866-024-03577-x (PMC11520699; doi:10.1186/s12866-024-03577-x)
Supplement: Supplementary file 1 — Supplementary Material 1 [file 12866_2024_3577_MOESM1_ESM.docx]

**Synergistic Potential of Leu_10_-teixobactin and Cefepime Against Multidrug-Resistant *Staphylococcus aureus***

**Augustine Jing Jie Koh^1,2,4^, Maytham Hussein^1,2^, Varsha Thrombare^2^, Simon Crawford^3^, Jian Li^4^, Tony Velkov^1,2^**

^1^Department of Biochemistry and Pharmacology, School of Biomedical Sciences, Faculty of Medicine, Dentistry and Health Sciences, The University of Melbourne, Parkville, VIC 3010, Australia

^2^Monash Biomedicine Discovery Institute, Department of Pharmacology, Monash University, Clayton, Victoria 3800, Australia

^3^Monash Biomedicine Discovery Institute, Department of Biochemistry and Molecular Biology, Monash University, Clayton, Victoria 3800, Australia

^4^Monash Biomedicine Discovery Institute, Department of Microbiology, Monash University, Clayton, Victoria 3800, Australia

**Corresponding authors:** tony.velkov@monash.edu OR jian.li@monash.edu

**Keywords:** Leu_10_-teixobactin, cefepime, methicillin-resistant *Staphylococcus aureus* (MRSA), synergistic activity, biofilm inhibition, β-lactam potentiation

**Short Title:** Synergistic Activity Against MRSA: Leu_10_-teixobactin and Cefepime

**Supplementary Table S1.** Changes in colony counts of the L_10_TXB-CEF combinations at 2, 6, and 24 h. The green background indicated synergy (≥2-log_10_CFU-decrease in CFU/mL with the combination relative to the more active antibiotic L_10_TXB with the population of viable bacteria to be ≥2-log_10_CFU below the starting inoculum) while the yellow background indicated additivity (>1-2-log_10_CFU-decrease compared to the most active component). NA – Not Assessed. t – time point at which bacterial counts is observed post-treatment. θ – time point 0 h where initial inoculum is log_10_CFU ~6.0.

| Strain | Time (h) | Δlog_10_CFU = log_10_CFU_t_ – log_10_CFU_θ_ | | | | | |
| --- | --- | --- | --- | --- | --- | --- | --- |
|  |  | L_10_TXB (0.25×MIC) | L_10_TXB (0.5×MIC) | CEF (0.5×MIC) | CEF (≤0.125×MIC) | L_10_TXB (0.25×MIC) + CEF (0.5×MIC) | L_10_TXB (0.5×MIC) + CEF (≤0.125×MIC) |
| ATCC™29213 | 2 | 0.02 | NA | 0.51 | NA | -0.30 | NA |
|  | 6 | 0.23 |  | 1.06 |  | -1.33 |  |
|  | 24 | 2.23 |  | 2.28 |  | -3.88 |  |
| JKD 6159 | 2 | -0.36 |  | 0.28 |  | 0.00 |  |
|  | 6 | -0.58 |  | -0.49 |  | -1.30 |  |
|  | 24 | 3.53 |  | 2.76 |  | -2.93 |  |
| ATCC™43300 | 2 | NA | -0.87 | NA | -0.15 | NA | -0.15 |
|  | 6 |  | -0.96 |  | -0.05 |  | -1.14 |
|  | 24 |  | 1.38 |  | 2.40 |  | -2.07 |
| ATCC™700698 | 2 |  | -0.45 |  | 0.42 |  | -0.67 |
|  | 6 |  | -1.62 |  | 2.54 |  | -1.82 |
|  | 24 |  | 1.50 |  | 3.24 |  | -1.20 |
| ATCC™700699 | 2 |  | 0.21 |  | 0.08 |  | 0.01 |
|  | 6 |  | 0.83 |  | 0.73 |  | -0.27 |
|  | 24 |  | 3.73 |  | 2.97 |  | 3.28 |

**Supplementary Table S2.** List of primers used for RT-qPCR analysis of the selected genes (1-4).

| Gene | Function | Primers (5’ 🡪 3’) | T_m_ (°C) |
| --- | --- | --- | --- |
| *16s rRNA* | Housekeeping | F: GGG CTA CAC ACG TGC TAC AA | 63.8 |
|  |  | R: GTA CAA GAC CCG GGA ACG TA | 63.6 |
| *icaA* | Initial bacterial adhesion and aggregation for biofilm production | F: ACA CTT GCT GGC GCA GTC AA | 69.4 |
|  |  | R: TCT GGA ACC AAC ATC CAA CA | 64.1 |
| *atlA* | Peptidoglycan breakdown for daughter cell segregation | F: AAC AGC ACC AAC GGA TTA C | 60.0 |
|  |  | R: CAT AGT CAG CAT AGT TAT TCA TTG | 57.1 |
| *sarA* | Regulator of biofilm production and virulence | F: GTT ATC AAT GGT CAC TTA TGC | 56.1 |
|  |  | R: CTT GTG GTT GTT TGT AGT TT | 54.2 |
| *mecA* | β-lactam resistance | F: GTA GAA ATG ACT GAA CGT CCG ATA A | 64.0 |
|  |  | R: CCA ATT CCA CAT TGT TTC GGT CTA A | 68.3 |
| *pbp2* | Peptidoglycan crosslinking | F: CGT TTC TAC GAA CAT GGC GCA C | 70.5 |
|  |  | R: GGC ACC TTC AGA ACC AAA TCC ACC A | 74.2 |

**Supplementary Figure S1.**

**(A).** Structure of Leu_10_-Teixobactin

**(B).** HPLC and MS Data of Leu_10_-Teixobactin


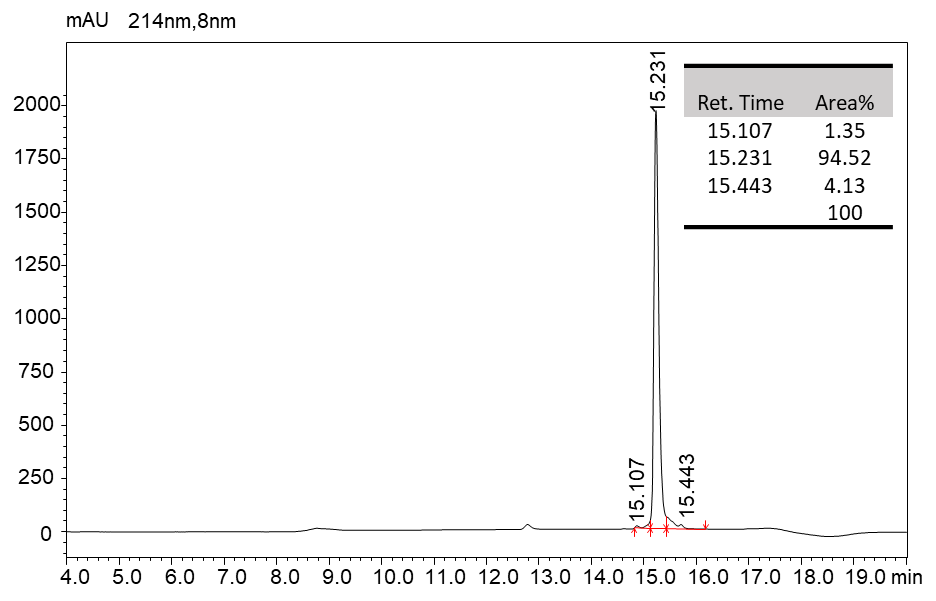


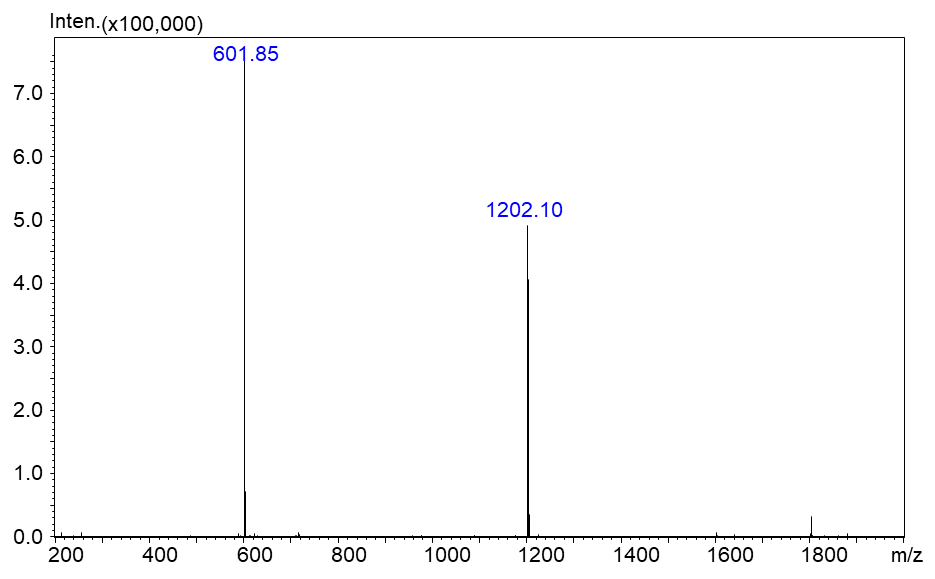


**References**

1. Sharafi T, Ghaemi EA, Rafiee M, Ardebili A. Combination antimicrobial therapy: in vitro synergistic effect of anti-staphylococcal drug oxacillin with antimicrobial peptide nisin against Staphylococcus epidermidis clinical isolates and Staphylococcus aureus biofilms. Annals of Clinical Microbiology and Antimicrobials. 2024;23(1):7-.

2. He X, Zhang W, Cao Q, Li Y, Bao G, Lin T, et al. Global Downregulation of Penicillin Resistance and Biofilm Formation by MRSA Is Associated with the Interaction between Kaempferol Rhamnosides and Quercetin. Microbiology Spectrum. 2022;10(6).

3. Noumi E, Merghni A, Alreshidi M, Del Campo R, Adnan M, Haddad O, et al. Phenotypic and Genotypic Characterization with MALDI-TOF-MS Based Identification of Staphylococcus spp. Isolated from Mobile Phones with their Antibiotic Susceptibility, Biofilm Formation, and Adhesion Properties. International Journal of Environmental Research and Public Health. 2020;17(11):3761-.

4. Chung PY, Chung LY, Navaratnam P. Identification, by gene expression profiling analysis, of novel gene targets in Staphylococcus aureus treated with betulinaldehyde. Research in Microbiology. 2013;164(4):319-26.
